# Immune History and Influenza Vaccine Effectiveness

**DOI:** 10.3390/vaccines6020028

**Published:** 2018-05-21

**Authors:** Joseph A. Lewnard, Sarah Cobey

**Affiliations:** 1 Center for Communicable Disease Dynamics, Department of Epidemiology, Harvard TH Chan School of Public Health, Boston, MA 02115, USA; jlewnard@hsph.harvard.edu; 2 Department of Ecology and Evolution, University of Chicago, Chicago, IL 60637, USA

**Keywords:** vaccine effectiveness, repeat vaccination, original antigenic sin, test-negative design, seasonal influenza vaccine, universal influenza vaccine, imprinting

## Abstract

The imperfect effectiveness of seasonal influenza vaccines is often blamed on antigenic mismatch, but even when the match appears good, effectiveness can be surprisingly low. Seasonal influenza vaccines also stand out for their variable effectiveness by age group from year to year and by recent vaccination status. These patterns suggest a role for immune history in influenza vaccine effectiveness, but inference is complicated by uncertainty about the contributions of bias to the estimates themselves. In this review, we describe unexpected patterns in the effectiveness of seasonal influenza vaccination and explain how these patterns might arise as consequences of study design, the dynamics of immune memory, or both. Resolving this uncertainty could lead to improvements in vaccination strategy, including the use of universal vaccines in experienced populations, and the evaluation of vaccine efficacy against influenza and other antigenically variable pathogens.

## 1. Introduction

Inactivated and live attenuated vaccines are the key public health tools against influenza. Since vaccines must be re-formulated semi-annually to counteract antigenic changes in influenza viruses, the low effectiveness of influenza vaccines has traditionally been attributed to mismatch between vaccine strains and circulating strains [1,2,3,4]. The sometimes complicated task of predicting antigenic match can be exacerbated by mutations acquired during manufacture. This can lead to another form of mismatch and is a clear cause of reduced effectiveness [5].

However, even when the match between the vaccine and circulating strains is good, vaccines may not be perfectly protective in healthy, non-aged subjects. Even for pathogens that do not rapidly evolve to escape immunity, vaccines rarely confer complete protection from infection in all people, potentially due to variation in vaccine immunogenicity and variation in individuals’ responsiveness [6]. The temporal and demographic patterns underlying estimates of influenza vaccine effectiveness suggest a more complicated story, albeit one that has proven difficult to untangle in the presence of both scientific uncertainties and limitations to the epidemiological methods used to measure influenza vaccine performance. These patterns, coupled with increasing insight into the immunology of the response, suggest that improvements to influenza vaccines may be constrained without a better understanding of the effect of past influenza exposures on host responses to vaccination. This understanding could improve not just seasonal vaccine effectiveness but also predictions of season severity, viral evolution, and the design of universal vaccines for populations with prior influenza exposure.

## 2. What Do We Expect, and What Do We See?

**Figure 1:**
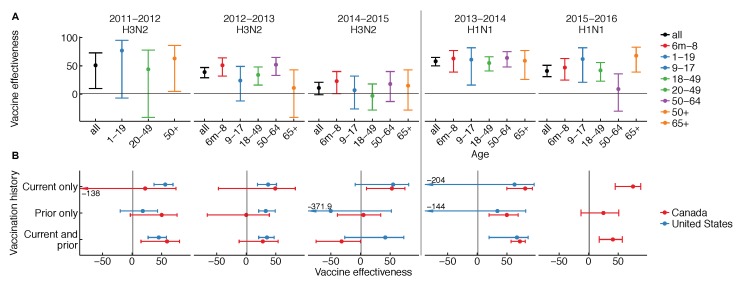
Vaccine effectiveness from the U.S. and Canada (**A**) overall and by age group and (**B**) by recent vaccination history. Comparisons must be made with caution due to differences in study design and study population. Please see Table A1 in the Appendix A for details.

The effectiveness of influenza vaccines is unusual in three ways: it appears to vary (1) from season to season, (2) by age group from season to season, and (3) with vaccination history. Patterns in vaccine effectiveness are not consistent among H1N1, H3N2, and influenza B over time, and are rarely ascertained from “gold-standard” randomized, placebo-controlled trials. Variation in vaccine effectiveness by season, age, and vaccination history have each been observed in multiple years and populations (Figure 1) [1,7,8,9,10].

These patterns depart from traditional expectations for vaccines. If influenza did not evolve antigenically, vaccine-induced protection did not vary among age groups or wane in time, and coverage were constant and unassociated with individual risk factors, effectiveness would stay the same from year to year. It also would not vary by age. Influenza, of course, evolves antigenically over time, leading to variable vaccine effectiveness by year. In general, we would expect that even with this relaxed assumption, “null” variation expected to arise in age-stratified estimates of vaccine effectiveness should not be systematically associated with other factors.

We see instead that effectiveness varies not just over time, but also by age over time and between more and less frequent vaccinees in some seasons (Figure 1) [10]. The latter two observations are hard to explain. In at least one instance, differences in effectiveness between age groups have varied from one season to the next. This is clearest for the 2015–2016 season, which was dominated by H1N1, wherein stark differences in vaccine protection were noted for older middle-aged adults. The pattern was observed not just in the United States [11] but also in Canada [12]. For H3N2, the story is unclear, but interim vaccine effectiveness estimates from the past two seasons hint at variation (Figure 1 and [13,14]). In some seasons, vaccine effectiveness was lower in people immunized in both the current and previous season compared to people immunized in the current season only (Figure 1) [10]. Analyses spanning multiple seasons have also found a negative relationship between vaccination frequency and effectiveness, e.g., in patients at least nine years old in Marshfield, Wisconsin, from the 2004–2005 to 2012–2013 seasons [15], and in children 9–18 years old on Kamigoto Island, Japan, from the 2010–2011 to 2013–2014 seasons [16]. Infamously, vaccine effectiveness estimates from Canada have sometimes appeared negative in the most heavily vaccinated groups [17], and in Hong Kong and Canada, negative vaccine effectiveness was reported for the trivalent seasonal vaccine against pandemic H1N1 in 2009 [18,19], a pattern not found elsewhere [20,21]. Certainly, understanding what causes the vaccine to appear to lose effectiveness in some age groups and some seasons, and to lose effectiveness in the same people over time, is an important challenge.

## 3. Are Our Measures of Vaccine Effectiveness Part of the Problem?

A first consideration when accounting for variation in estimates of vaccine effectiveness is whether observed differences in influenza endpoints between vaccinated and unvaccinated persons necessarily measure the immunizing effect of a vaccine. What we often want to know is the reduction a vaccine confers to an individual’s susceptibility to influenza infection or influenza-caused illness, measured relative to the same individual’s susceptibility had he or she not received the vaccine. Epidemiologists often refer to this as “vaccine efficacy” (in the context of randomized studies), “vaccine effectiveness” (in observational studies of individual outcomes), or the vaccine “direct effect” [22], and varyingly measure it as one minus the risk ratio, hazard ratio, or odds ratio of an influenza clinical endpoint given vaccination depending upon study design (Appendix B). Because the effect of interest is usually the same for all three terms [23], we will use the term “VE” (referring to vaccine effectiveness) throughout. As discussed, the protective effect of influenza vaccines can reasonably be expected to vary within and between populations. Thus, we may be interested in estimates that are stratified by susceptibility or responsiveness class. To obtain an average VE for any population, we could vaccinate randomly and compare the rate at which influenza infections occur in the vaccinated and unvaccinated groups, or the cumulative incidence of infections among vaccinated and unvaccinated persons.

Most VE estimates, however, do not come from randomized trials, nor do they closely track the rate of influenza infection in vaccinated and unvaccinated people; trials cannot practically be undertaken in advance of the influenza season for vaccines that are updated semi-annually, and trials pose ethical challenges in settings such as the U.S., Canada, and Australia, where annual vaccination is recommended for all individuals over six months old. Since 2004, the most common method for estimating the effectiveness of seasonal influenza vaccines has been the test-negative design (TND). In contrast to randomized trials and observational cohort studies that follow vaccinated and unvaccinated individuals prospectively to compare influenza outcomes, the TND compares the prevalence of prior vaccination among individuals who seek care for influenza-like illness and receive a positive or negative outcome of a laboratory test for influenza infection. In this way, the TND resembles a “retrospective cohort” design [24] or a case-control study enrolling controls with an “imitation” disease [25]. By conditioning on the healthcare-seeking behavior of individuals, the TND thus tries to adjust for bias imposed by the fact that individuals who seek influenza vaccination may differ from those who do not seek influenza vaccination in their likelihood of seeking medical attention for acute respiratory infection [23].

The ability of TND studies to recover reliable VE estimates under a set of ideal assumptions led to enthusiasm about uses of the TND as a basis for policy-making [23,26,27]. However, both historically-recognized [24,28,29] and newly-appreciated biases arising under the TND have received increasing attention in recent years [30,31,32,33]. One recent evaluation illustrates that VE estimates from the TND may be biased to the point that vaccines that are actually protective can appear to facilitate infection [34]. This increasing scrutiny of the TND has been reflected in debate about its validity as a basis for causal inference [35,36], and about the appropriateness of decisions premised on TND studies [37,38]. In 2018, a determination was made not to update the Cochrane Reviews database on influenza vaccine effectiveness with results from TND studies [39,40,41]. The potential for biases draws into question whether variation in VE estimates is necessarily attributable to factors like immune history, or can instead be explained by confounding associations between individuals’ susceptibility and likelihood to seek vaccination, as well as more fundamental biases affecting TND studies.

Perhaps the most important limitation of the TND arises from its observational nature, insofar as individuals’ decisions to vaccinate may be associated with other factors influencing their exposure or susceptibility to influenza. For instance, older individuals who vaccinate may be generally healthier [42], and vaccination is strongly encouraged among many high-risk groups, including educators and healthcare workers. Studies usually have limited ability to resolve these differences in susceptibility and exposure risk; whereas, test-negative studies of influenza vaccine effectiveness have not traditionally included extensive covariate information, the availability of such data in recent test-negative studies of rotavirus vaccine effectiveness (e.g., [43]) has provided added insight. Although the functional forms of bias under the TND are not universally amenable to correction by controlling for covariates, odds ratio (OR) estimates stratified by risk status may recover VE estimates closer to the true effect, albeit at the expense of statistical power [34]. Because VE estimates under the TND are measured relative to the association of vaccination with a test-negative control endpoint, bias relating to this source of confounding occurs if factors associated with vaccination status have a specific impact on risk for influenza or on other chronic or infectious causes of acute respiratory symptoms. This topic has received attention in previous assessments of the theoretical basis of the TND for influenza vaccine effectiveness [30,31,33,34]. Other uses of the TND—for instance, to distinguish VE against vaccine-serotype pneumococcal infection via comparisons against non-vaccine serotype infection [24]—may more reasonably satisfy this condition than influenza studies, given the diverse etiologies that may be represented among test-negative individuals.

These concerns about confounding determinants of both susceptibility and individuals’ likelihood to vaccinate have particularly acute implications in studies of the effect of repeated vaccination. People who choose to be vaccinated tend to be vaccinated in other seasons, opening the possibility for confounding by previous vaccination as well as infection history in the relationship between current-season vaccination and influenza. Stratifying VE based on vaccination history can partially adjust for such differences [34]; however, unmeasured confounding variables between individuals’ histories of vaccination and previous influenza infection will limit the transportability of VE estimates for populations with differing past influenza exposures. An ideal study would stratify VE according to past vaccination, exposure, and infection history in order to isolate the effect of vaccination on susceptibility to infection within the same season. However, the reliance on retrospectively collected data under the TND makes it difficult to define such counterfactual comparisons.

A second and more fundamental class of biases, which affects the TND as well as randomized controlled trials [44,45,46], arises because measures of statistical association may not capture the vaccine effect on susceptibility to infection or disease. Under an ideal scenario of randomized vaccine uptake, the odds ratio of vaccination among test-positive versus test-negative subjects recovers the proportion of individuals protected by an “all-or-nothing” vaccine effect [28,34]. That is, some fraction of recipients respond to the vaccine and are perfectly protected from infection, while the susceptibility of other recipients is unaffected by vaccination. In contrast, the odds ratio will not accurately recover a “leaky” vaccine effect, under which vaccine responders have incomplete protection. For influenza, vaccine protection is arguably leaky, in that people who exhibit at least a four-fold increase in titer from vaccination, the traditional marker of vaccine immunogenicity and “responder” status, can still be infected. The magnitude of biases that result from this condition depends on individuals’ total risk of having acquired influenza by the time they enter the study—for instance, due to high rates of transmission in the population, or the assessment of VE either early or late in the season. Thus, findings of variation across populations and over time in VE estimates may not necessarily indicate differences in vaccine performance, drawing into question whether observations of reduced protection by the end of an influenza season [47] necessarily indicate vaccine waning.

The potential for design-level sources of epidemiological bias to contribute to variation in VE estimates underscores the need for improved methods to understand factors influencing differential protection among individuals. Because there are few head-to-head comparisons between estimates from the TND and other studies within the same population [48], and because biases affecting the TND may also affect other studies of influenza vaccines, the magnitude of bias in current studies remains unknown. Nonetheless, several lines of evidence discussed below suggest that the distinctive patterns of VE in populations over time could also arise from genuine differences in the ability of the vaccine to protect different subpopulations from medically attended influenza infections. Although these findings do not exclude flaws in epidemiological study designs, agreement between these lines of experimental evidence and VE estimates lends support to the hypothesis that immune history shapes the vaccine response.

## 4. Biological Explanations: Original Antigenic Sin

One of the oldest theories in influenza has recently re-emerged as a potential explanation for low vaccine effectiveness, differences in vaccine effectiveness between age groups over time, and also for reduced effectiveness in repeat vaccinees. Both immunological and epidemiological research lend credibility to the hypothesis.

The theory of original antigenic sin (OAS) holds that the antigens on influenza strains encountered in childhood permanently shape the antibody response, such that antibodies recognizing conserved antigens are boosted upon exposure to related strains, and often at the expense of responses to novel antigens. Early studies demonstrated that, after immunization with H1N1, people produced antibodies that cross-reacted with historic strains, but only if subjects were old enough to have been infected with these strains in childhood [49,50]. Thus, although subjects from all age groups responded to the vaccine strain, the quality or cross-reactivity of their response depended on their presumed infection history. Adults and children today still show different patterns of cross-reactivity after influenza vaccination [51]. There is also strong evidence in humans [52,53,54,55,56,57,58,59,60] and animal models [61,62] that memory B cells, presumably specific to conserved epitopes, are commonly reactivated in later influenza exposures. One implication is that cohorts differ in which sites are targeted by protective antibodies, and these sites are partly determined by past exposures; thus, as the virus mutates its epitopes, different subpopulations—defined directly by their antibody specificities, and indirectly by exposure histories—might lose protection at different times [63]. OAS might be involved in susceptibility on another scale. Gostic et al. observed protection from severe disease and death caused by avian subtypes whose hemagglutinin surface proteins are more related to the seasonal influenza subtypes from childhood [64]. Gagnon et al. found that individuals born during the H2N2 pandemic had increased susceptibility to mortality from pandemic H1N1 in 2009 and 2013–2014 [65].

Like natural infection, vaccination could interact with OAS and affect protection. Twenty years ago, Smith et al. proposed that variable vaccine efficacy arises from the relationships between the cross-reactivities of the past vaccine strain, current vaccine strain, and circulating strain [66]. In their model, when the past and current vaccine strains are similar, the current vaccine boosts responses that cross-react with the previous vaccine strain. If few of these antibodies react well with circulating strains, vaccine effectiveness will be low. In contrast, in people who were not previously vaccinated, or if the previous vaccine strain cross-reacts minimally with the current vaccine strain, the effectiveness of the current vaccine (even if not a great match to the circulating strain) will be higher. This “negative interference” between vaccines arises from reactivation of the memory responses that are conserved between vaccine strains at the expense of responses to new antigen. Although the model uses an abstracted shape space to represent antigenic relationships between strains, its form is consistent with the basic—albeit slightly ambiguous—dynamics of OAS. Here, however, the sin is not strictly “original” and can arise from recent vaccination.

The detail of recent VE studies suggests observations that are consistent with this model, which might explain variable VE from year to year and perhaps reduced VE in repeat vaccinees. Skowronski et al. have suggested that negative interference could explain low vaccine effectiveness in two recent H3N2 seasons [67]. In Canada in 2012–2013, VE trended higher in people vaccinated only in that season compared to people vaccinated in the current and previous season. This trend was not apparent in the US, but in partial support of the negative interference model, an analysis of responses in a heavily immunized adult cohort suggested that the vaccine boosted only cross-reactive epitopes [68]. However, the boosted titers also appeared to cross-react just as strongly with circulating strains, which leaves open the question of why VE was so low. In 2014–2015, the addition of a glycosylation site in circulating viruses led to very low vaccine effectiveness [69]. Due to mutations in manufacturing, the H3N2 component of the egg-grown vaccine has remained unglycosylated at this site since then. Tellingly, adults immunized in the 2016–2017 season with the unglycosylated, egg-adapted strain mounted responses that were focused on the unglycosylated site, which should confer little protection against infection [5]. Responses to the unglycosylated site were especially high in adults who had been immunized in each of the past two seasons with the unglycosylated strain, suggesting again that the vaccine mostly boosts pre-existing responses.

The dynamics of OAS might also explain differences in estimates of VE by age over time. The strength of the response to the unglycosylated site on recent H3N2 varied by age in adults, and was generally higher in adults who had seen a similar unglycosylated site in childhood [68]. This suggests the vaccine boosted not only responses from past vaccines, but also responses whose specificity was shaped by early exposures. More dramatic evidence comes from the 2015–2016 season, which was dominated by H1N1. In the U.S. and Canada, VE against H1N1 dropped noticeably in older and middle-aged adults (Figure 1). In Canada, it was also shown to be lower in repeat vaccinees [12]. Two studies suggested that the strong age-associated reduction in VE was consistent with OAS-like effects. In that season, circulating H1N1 acquired a glycosylation site [11,12], there were strong correlations between birth year and specificity to this site, and previous work had shown this specificity arose by OAS [70]. Although the possible influence of other age-related factors has yet to be conclusively ruled out, these studies collectively suggest that even in adults, the response to vaccination might interact with memory established decades earlier to generate age-dependent patterns in protection. Such cohort-specific differences in protection provide a natural discontinuity design for causal inference, isolating early influenza antigenic exposures as a potential factor underlying sharp differences in protection between age groups.

The influence of OAS on response to vaccination, strength of protection, and VE estimates remains complicated, however. It is unclear how much negative interference between old and new responses there really is. Early work on OAS showed that, regardless of age and immune history, vaccine recipients could develop high titers to any historic immunizing strain [49,50]. Furthermore, vaccination did not always merely boost antibody responses that cross-reacted with childhood strains but could sometimes induce responses unique to the immunizing strain [49,50]. Vaccination could also eventually lead to a persistent shift in the cross-reactivity profile (or “antibody landscape” [59]) to a given vaccine strain [50]. In contrast, the negative interference model and recent studies of the vaccine response in adults hint at less tendency to target new sites [5,11,12,68]. Adjuvants are known to attenuate the effects of OAS [71,72], and this may explain potential discrepancies between recent patterns and early studies, which relied on adjuvants [50] or multiple immunizations in a short period [49,50]. Another missing piece is that for OAS to explain changes in VE by age over time, antibodies of different specificities should vary in their protectiveness. This is plausible. Cross-reactive antibodies to influenza are extremely common [52,73,74], but not all cross-reactive responses are equally protective [75]. Although there has been extensive work correlating hemagglutinin and neuraminidase titers to protection (e.g., [76]), the relationships between fine-scale specificity to particular epitopes, antibody concentration, antibody isotype, antibody avidity, and protection are not well understood.

## 5. Other Biological Explanations

Another curiosity of the influenza vaccine is that some people appear not to respond to it. This is suggested partly and indirectly by the low vaccine effectiveness, but it also appears in standard tests of the vaccine response. For instance, less than 20% of children and adults had at least a four-fold titer rise against H3N2 and influenza B after immunization [77]. Although there is evidence for a ceiling effect, whereby people with initially high titers have smaller boosts [78], many non-responders start with low or moderate titers [68,79]. One possibility is that people are responding, but these responses are simply not being measured (e.g., if most antibodies target the neuraminidase [57]). Another is that there are significant differences in individual capacities to respond [6]. Reduced responsiveness has been associated with age-associated thymic involution [80], obesity [81], statin use [82,83] (but see [84]), lack of cytomegalovirus infection (in the young) [85], presence of cytomegalovirus infection (in the old) [86], male sex [87], and other factors, especially ones related to baseline levels of inflammation [6,88].

These factors might explain differences in VE between older and younger individuals. It is less obvious how they could explain substantial interannual variation in VE by themselves, although some suggest opportunities for other pathogens to affect VE. It is also not obvious how these factors would create variation in the relative VE by age over time.

## 6. Future Directions

A recent theoretical study showed that, if antigenic mismatch from evolution were the only cause of reduced vaccine effectiveness, it should not be hard to prevent transmission at coverages below those of the U.S. [89]. Improving the effectiveness of seasonal influenza vaccines remains a tantalizing prospect, but it requires overcoming two main challenges: assessing the contributions of bias and biology to estimates of effectiveness.

Understanding the basis of low VE will be easier if VE itself is measured accurately. A head-to-head comparison of VE estimates from TND and a randomized trial in a representative population could provide insight into the magnitude of bias and reliability of past estimates. Identifying the bias’s source might require further investigation, such as a prospective study. Although it is now common to stratify subjects by vaccination history, it would also be useful to measure associations between voluntary vaccination and infection risk, symptom severity, and propensity to seek medical attention or testing, and to identify immunological signatures of infection history. These data would also greatly inform knowledge of basic influenza epidemiology, including estimates of incidence.

Several approaches could help untangle the role of immune history in vaccine effectiveness. One of the simplest would be to evaluate the antigenicity of candidate vaccine strains using not only ferret antisera, as most often practiced, but also representative human sera, which may provide early clues into cross-sectional differences in responsiveness. Differences between human and ferret cross-reactivity profiles were used to argue for covert antigenic mismatch in 2013–2014 [70] and against reported mismatch in 2012–2013 [68]. It would be useful to know if antigenicity inferred from human sera better predicts vaccine effectiveness than antigenicity inferred from ferret sera. At the other end of the spectrum of difficulty, understanding in fine detail the immune correlates of vaccine responsiveness and protection could illuminate the roles of OAS and other mechanisms in vaccine effectiveness. Data from virtually any observational longitudinal studies could be valuable. More comprehensive measures of anti-influenza antibody responses could show if there are true non-responders, or if some studies are simply failing to detect immunodominant responses to sites farther from the hemagglutinin’s receptor binding domain. Because the hypothesized mechanisms of low vaccine effectiveness involve complex interactions between infections, vaccinations, competing B and T cell populations specific to different epitopes, waxing and waning titers, and age-related changes in infection risk, interpretation of the data will not be straightforward. Dynamical hidden Markov models are especially useful for identifying interactions involving time lags, nonlinearities, and unobserved states (e.g., [60]). These less traditional epidemiological approaches would be complemented by recent breakthroughs in experimental immunology, including high-throughput single-cell methods that can deconstruct the components of affinity maturation and immune response in detail. Effectively, identifying the role of immune history in vaccine effectiveness requires modeling processes across several scales.

## 7. Conclusions

Davenport et al. cautioned in 1957 that strong memory to childhood strains would make it difficult to immunize against the “virus of the year” with all its “minor antigenic caprice”, but noted that adjuvanted polyvalent vaccines at multiannual intervals might recapitulate the broad responses and protection eventually acquired with age [50]. The aim of universal vaccines is to induce broad responses, typically to a few conserved epitopes on the stalk. It remains unclear how history will modulate the short- and long-term effectiveness of these vaccines: the animal models on which the promising results are based have no prior immunity to influenza. It is plausible that adults immunized with universal vaccines might sometimes target familiar non-stalk epitopes, such as the neuraminidase, and some of these antibodies may be less broadly protective. Diverse exposure histories could lead once again to variation in vaccine effectiveness between populations, which could invite escape mutations.

The challenges associated with influenza vaccines are exciting because they have the potential to reveal fundamental features of immune memory against antigenically variable pathogens, which remain a large source of morbidity and mortality and prime targets for vaccine development. This immune memory is a powerful selective force, and understanding the ways in which vaccination shapes it could also improve forecasting. Furthermore, understanding the strengths and limitations of current study design will be vital not just for influenza vaccines but also for vaccines against other antigenically complex pathogens, for which efficacy must be assessed in a dynamic background of immune history [90].

## Abbreviations

The following abbreviations are used in this manuscript:

OAS	Original antigenic sin
OR	Odds ratio
TND	Test negative design
VE	Vaccine efficacy or effectiveness (defined here as the vaccine direct effect on susceptibility to infection and/or disease)

## Appendix A

**Table A1:** Sources of data for Figure 1. Except where noted below, vaccine effectiveness is shown only for the dominant circulating subtype each season.

Season	VE Type	Notes	Source
2011–2012	Age	All ages	Canada [91]
	Vaccination history	≥9 years, all flu	U.S. [92]
		≥2 years	Canada [91]
2012–2013	Age	All ages	U.S. [93]
	Vaccination history	≥9 years	U.S. [93]
		≥9 years	Canada [67]
2013-2014	Age	≥6 months	U.S. [94]
	Vaccination history	≥9 years	U.S. [95]
		≥2 years	Canada [96]
2014–2015	Age	≥6 months	U.S. [97]
	Vaccination history	≥18 years	U.S. [98]
		≥2 years	Canada [17]
2015–2016	Age	≥6 months	U.S. [97]
	Vaccination history	≥9 years	Canada [12]

## Appendix B. Measuring VE against Influenza

Several different approaches are commonly taken to measure the extent to which vaccination protects recipients against infection and disease. When participants are enrolled in a study before an influenza season begins—as might be possible in a trial or a prospective cohort study—VE can be measured via the reduction in the hazard ratio (HR) or risk ratio (RR) of influenza over the study period:

$${\hat{VE}}_{HR}=1-HR=1-\frac{\left(\frac{\mathrm{Cases}\;\mathrm{among}\;\mathrm{the}\;\mathrm{vaccinated}}{\mathrm{Vaccinated}\;\mathrm{person}-\mathrm{years}\;\mathrm{at}\;\mathrm{risk}}\right)}{\left(\frac{\mathrm{Cases}\;\mathrm{among}\;\mathrm{the}\;\mathrm{unvaccinated}}{\mathrm{Unvaccinated}\;\mathrm{person}-\mathrm{years}\;\mathrm{at}\;\mathrm{risk}}\right)},$$

or

$${\hat{VE}}_{RR}=1-RR=1-\frac{\left(\frac{\mathrm{Cases}\;\mathrm{among}\;\mathrm{the}\;\mathrm{vaccinated}}{\mathrm{Vaccinated}\;\mathrm{persons}\;\mathrm{at}\;\mathrm{risk}}\right)}{\left(\frac{\mathrm{Cases}\;\mathrm{among}\;\mathrm{the}\;\mathrm{unvaccinated}}{\mathrm{Unvaccinated}\;\mathrm{persons}\;\mathrm{at}\;\mathrm{risk}}\right)}.$$

If individuals’ decision to get vaccinated is unassociated with their risk of influenza exposure or susceptibility to infection and disease, ${\hat{VE}}_{HR}$ will recover unbiased estimates of either “leaky” or “all-or-nothing” protection, whereas ${\hat{VE}}_{RR}$ may underestimate “leaky” protection when evaluated late in the season or in a population exposed to high transmission rates [28].

However, most influenza VE estimates are generated under study designs where the total population of vaccinated and unvaccinated persons is not known. In such instances, case-control or test-negative study designs may be undertaken to estimate VE from the exposure odds ratio (OR) of vaccination associated with influenza case status. In a case-control study selecting control individuals from the community not known to have experienced influenza,

$${\hat{VE}}_{OR}=1-OR=1-\frac{\left(\frac{\mathrm{Vaccinated}\;\mathrm{influenza}\;\mathrm{cases}}{\mathrm{Unvaccinated}\;\mathrm{influenza}\;\mathrm{cases}}\right)}{\left(\frac{\mathrm{Vaccinated}\;\mathrm{community}\;\mathrm{controls}}{\mathrm{Unvaccinated}\;\mathrm{community}\;\mathrm{controls}}\right)}.$$

For the assumptions under which case-control and test-negative study estimates are interpreted to measure vaccine effectiveness, ${\hat{VE}}_{OR}={\hat{VE}}_{RR}$ [23,26]. However, measures from the OR will only provide valid estimates of “leaky” protection early in the influenza season or in low-transmission settings [28,34]. In addition, it is possible that estimates from a case-control or prospective cohort study may be biased by the fact that healthcare-seeking behavior drives certain individuals to receive the influenza vaccine as well as to receive treatment when infected. Under the test-negative design (TND), ${\hat{VE}}_{TND}$ is calculated by comparing the exposure odds ratio of vaccination among individuals seeking care for acute respiratory infection who receive a positive or negative outcome of an influenza diagnostic test:

$${\hat{VE}}_{TND}=1-OR_{TND}=1-\frac{\left(\frac{\mathrm{Vaccinated}\;\mathrm{influenza}-\mathrm{positive}\;\mathrm{cases}}{\mathrm{Unvaccinated}\;\mathrm{influenza}\;\mathrm{cases}}\right)}{\left(\frac{\mathrm{Vaccinated}\;\mathrm{influenza}-\mathrm{negative}\;\mathrm{cases}}{\mathrm{Unvaccinated}\;\mathrm{influenza}-\mathrm{negative}\;\mathrm{cases}}\right)}.$$
